# Implementation of the modified four‐step approach method for teaching echocardiography using the FATE protocol—A pilot study

**DOI:** 10.1111/echo.14128

**Published:** 2018-08-26

**Authors:** Agnieszka Skrzypek, Tomasz Górecki, Paweł Krawczyk, Mateusz Podolec, Grzegorz Cebula, Konrad Jabłoński, Marta Szeliga, Michał Nowakowski

**Affiliations:** ^1^ Faculty of Medicine Department of Medical Education Jagiellonian University Medical College Cracow Poland; ^2^ Department of Anesthesiology and Intensive Care Medicine Jagiellonian University Medical College Cracow Poland; ^3^ Coronary Heart Disease and Coronary Insufficiency Clinic with the Subdivision of Intensive Cardiological Supervision John Paul II Specialist Hospital in Cracow Cracow Poland

**Keywords:** echo, echocardiography, emergency imaging, FATE protocol, medical education, Peyton's four‐step approach, small group education, teaching

## Abstract

**Introduction:**

Peyton's four‐step approach is well‐known and commonly used in medical education. It is a practical and useful method which is simple to apply. The study presents the implementation of the modified four‐step approach method to teach how to perform the emergency echocardiographic assessment according to FATE (Focus‐Assessed Transthoracic Echo) protocol. The aim of the study was to determine the feasibility and utility of this method FATE protocol teaching.

**Design:**

We collected students' feedback relating to perception of this way of teaching. Based on a semistructured interview conducted with the students, as well as an evaluation of the electronic survey, it has been demonstrated that the four‐step method is useful for teaching emergency echocardiographic assessment.

**Setting:**

One Polish medical school.

**Participants:**

The classes were run in small groups as part of an elective ultrasound course for the fourth‐ and fifth‐year students of the Faculty of Medicine of the Medical College. Twenty‐two students were trained.

**Results:**

Based on the opinions of the participants of the elective course and the teacher conducting the classes, which involved the use of the modified Peyton's four‐step method in teaching echocardiography in emergency cases according to the FATE protocol, it has been determined that the four‐step method is effective in imaging training. All participants claim that this method is clear and understandable. Advantages of the methodological approach: a slow‐motion demonstration by the instructor, accompanied by the commentary on the activities undertaken and practical exercises performed by the participants, learning through repetition, requirement of constant concentration.

**Conclusions:**

Peyton's approach allows to use of the class time in maximal extend by consolidating new information and facilitating memorization through adequate instructor guidance and observation of the training of the peer students and repetition of the skills acquired.

**Strengths and limitations of this study**

- This study assessed the feasibility and utility of the modified four‐step approach method in teaching of the emergency echocardiographic assessment according to FATE (Focus‐Assessed Transthoracic Echo) protocol.
- Teaching of the echocardiographic imaging in the small group is the most effective, so the conditions of the course influence on the strength of the study and also on the limitation (a few participants of the study).
- We analyzed participants' perspectives from only one medical course entitled “Ultrasound imaging in life‐threatening situations” and that limits the generalizability of our findings.

## 1 INTRODUCTION

At the Faculty of Medicine of the Medical College, an elective course is organized, entitled “Ultrasound imaging in life‐threatening situations.” This kind of training is addressed to interested students of the fourth year and fifth year of study. The elective class is conducted at the Department of Medical Education of the Medical College by university teachers with practical experience in ultrasound imaging. The aim of the training was to teach emergency patient ultrasound assessment according to FAST (Focused Assessment with Sonography in Trauma), EFAST (Extended Focused Assessment with Sonography in Trauma), BLUE (Bedside Lung Ultrasonography in Emergency), FEEL (Focused Echocardiographic Evaluation in Life Support), and FATE (Focus‐Assessed Transthoracic Echo) protocols.^1^, ^2^, ^3^, ^4^, ^5^, ^6^, ^7^, ^8^, ^9^

All the aforementioned protocols used in emergency cases are of high practical importance. They shorten the time between the first contact with a patient in life‐threatening conditions and the correct diagnosis followed with the appropriate treatment.^10^, ^11^, ^12^, ^13^, ^14^ Students have the opportunity to independently conduct imaging on a healthy volunteer—another course participant of the course. They can repeatedly practice to consolidate the acquired knowledge of the ultrasound techniques. Classes are run in a small group of students, which allows for the implementation of the modified four‐step Peyton's method to teach echocardiographic imaging in emergency cases according to the FATE protocol.^15^ Initially, Peyton's method was meant to be used in a situation in which there is only one student per one instructor.^15^ In 2014, Nikendei et al attempted to implement a modified Peyton's method for teaching clinical skills, where one instructor instructed several students.

This publication concerns the students' perception of the modified Peyton's four‐step method, used during the elective course exclusively to teach echocardiographic imaging according to the FATE protocol. The remaining elements of training included in the framework of the elective course were taught in a traditional manner, involving demonstration by the instructor followed by practical exercises performed by the students, without the use of the four‐step method.

### 1.1 The aim of the study

The aim of the study was to determine the feasibility of this method in teaching echocardiography in accordance with the FATE protocol and the perception of the method by students.

## 2 MATERIALS AND METHODS

Over the last 2 years, an elective course was organized for the fourth‐ and fifth‐year medical students to familiarize them with the imaging examination quite frequently performed in life‐threatening cases, often performed in emergency departments or admission room, as well as to allow the students to practice it. During the first half of 2017, a new learning tool was implemented to teach echocardiographic imaging in emergency cases, that is, a modified Peyton's four‐step method, described in the literature in the field of medical didactics as an effective way to teach practical skills.^16^ The method facilitates understanding, consolidation, and independent presentation of the new content and skills.

As part of the elective class, 22 students were trained. Three weeks before the planned classes, each of the students was granted access to materials for self‐preparation for classes. The materials included current literature in terms of the course content, as well as videos of pathological ultrasound images. The practical classes were preceded by a precourse test to assess the participant's knowledge at the onset of the course. Subsequently, the students practiced, divided into three groups. The course lasted 30 hours.

1st day of the course included lectures and getting to know the equipment. 2nd, 3rd, and 4th day included workshops.

Each day: I group trained FATE (Focused Assessed Transthoracic Echo) protocol, II group—BLUE (Bedside Lung Ultrasound in Emergency), and III group FAST (Focused Assessment with Sonography in Trauma). The groups changed the trained protocol (and teacher) each 60 minutes.

Third‐ and Fourth‐day students practiced also ultrasonographic assessment of main vessels. The students received from the teacher the FATE card that can be requested free of charge at http://www.fate-protocol.com and are available as a free application for iPhone and Android. While students trained first time the FATE protocol, they focused on basic views:

1. Subcostal four‐chamber
2. Apical four‐chamber
3. Parasternal long axis
4. Parasternal LV short axis

The next day students trained the extended FATE views:

1. Subcostal vena cava
2. Apical two‐chamber
3. Apical long axis
4. Apical five‐chamber
5. Parasternal short‐axis mitral plane
6. Parasternal aorta short axis

The next day students repeated basic and extended FATE views. The students were only instructed to take the specifics ultrasound windows and to identify characteristic anatomical structures without taking more complex measurements.

The modified Peyton's four‐step method, used during the class on echocardiography in emergency cases according to the FATE protocol, was presented to students at the beginning of the training. Having determined that the participants had actually understood the instructions on how to proceed in Peyton's method, the teacher moved on to practical training.

In accordance with the stated objective of this study, it aims at discussing the value and the educational benefits of using the modified four‐step method. Therefore, the actual echocardiogram performed in emergency cases (eg, in patients with significantly lowered blood pressure and reduced consciousness, when cardiovascular disease is suspected as the cause for the severe condition of the patient) is discussed only briefly, to make persons without any medical background understand the manner in which the classes are conducted.

As part of the elective course, echocardiographic examination was performed according to the FATE protocol on a healthy participant of the course.

The echocardiographic imaging according to the selected protocol requires the application of a dedicated probe to the appropriate sites of the body of the person examined. Knowledge of the probe locations (in order to capture the so‐called parasternal long‐ and short‐axis view and then apical four‐, three‐ and two‐chamber view of the heart, subcostal view) and ability to visualize the heart are essential for proper imaging. Apart from the scheme of the echocardiographic procedure, time needed for proper visualization is crucial, because the right diagnosis and the immediate treatment depend on it. The modified Peyton four‐step method ensures effective use of the course time.

### 2.1 Below, the course of training applying the modified Peyton four‐step method is described in detail

#### 2.1.1 The first step

The teacher provides real time presentation of a given procedure, in demonstration quality, without any commentary Students watch the presentation—at this stage, questions are not allowed.

#### 2.1.2 The second step

Second slower presentation of the procedure with a detailed overview. At this stage, students are allowed to ask questions.

#### 2.1.3 The third step

The instructor involves a student, asking him to comment on the actions taken by the teacher during third demonstration. The remaining participants observe it.

#### 2.1.4 The fourth

The student, who previously commented on the examination, performs the echocardiographic test in accordance with the FATE protocol by himself, commenting on his actions.

After the completion of the above four steps, another series begins, in which the student involved in the two last stages (steps 3 and 4) of the previous series assumes the role of the teacher. Thus, theoretically, every participant of the training could do and comment on the whole procedure.

The remaining elements of the imaging (ie, the ultrasound examination according to the FAST/EFAST, BLUE, and FEEL protocols, as well as cervical vascular imaging) were taught in a standard manner, in which the presentation with a commentary was followed by the participants of the class practicing on one another.

To meet the assumptions of this study and determine the significance of the four‐step method in teaching imaging, a semistructured interview and a survey were conducted with the elective course students. This publication takes into account the statements made verbally by the students, as well as the information obtained with the use of an anonymous evaluation questionnaire collected after the end of the course.

Semistandardized interview contained two kind questions:

1. Open questions
  - What are your general impressions of the course?
  - What do you think about the usefulness of the modified four‐step approach method in teaching of echocardiographic examination using the FATE protocol?
  - What do you think about learning by observation?
  - What do you think about learning through repetition?
  - What do you think about the instruction in the four‐step method?
  - What do you think about learning by teaching others?
  - Could you give the advantages and disadvantages of the modified Peyton's four‐step method in teaching echocardiography according to the FATE protocol?
2. Close and open questions
  - Are you satisfied with the course? Why?
  - This method is practical or no in your opinion? Why?
  - Is the modified four‐step approach method understandable?

## 3 RESULTS

Twenty‐two students participated in the elective course “Ultrasound imaging in life‐threatening situations”, aged between 23 and 26, 24 years on average, including five men and 17 women. The students eagerly practiced the new method as part of the elective course in echocardiography.

The publication shows the advantages of the modified Peyton's four‐step method in teaching echocardiography according to the FATE protocol. Therefore, the comments of the study participants concern exclusively those activities in the class in which the method was used.

The students participating in the elective course of emergency imaging had not been familiar with the modified Peyton's four‐step method. They declared that they (quote) “have never heard of it before.” During the partially structured interviews, the elective course participants expressed their positive opinions on the modified Peyton's four‐step method.

Quantitative analysis of the semistandardized interviews conducted with the students trained.

Topics and categories resulting from the interviews conducted with the students (trainees).

### 3.1 The students' positive statements

Understanding the new didactic method of Peyton's four steps.

#### 3.1.1 The methodological approach

##### Learning by observation

“Easier, because you can learn how to hold the probe and where it should be applied.”

“That's the easiest way of learning.”

##### Learning through repetition

“Multiple repetitions allow you to master the examination technique better and make it easier to remember.”

“It allows you to remember the correct order in which the examination is executed and the technique.”

“Holding the head appropriately becomes a habit during repeated exercise.”

##### The value of the received instruction in the four‐step method before the classes start

“The one‐time oral instruction was sufficient and allowed me to use the method during the training.”

“It was understandable.”

##### Learning by peer‐teaching

“You cannot switch off during lessons.”

“The four‐step method makes you concentrate on the examination.”

“Teaching others, we learn ourselves, and we remember for longer.”

#### 3.1.2 General impressions of the course and the newly introduced four‐step method

All the trainees decided that the method is (quote) “easy to understand.”

The students decided that this was “not only a comprehensible and clear method, but also one which facilitates learning the imaging examination.”

One of the participants expressed his opinion in the following fashion:

“Echocardiographic imaging (in emergency cases) is hard, but owing to Peyton's four‐step method it is easier to take it in.”

Other students expressed their opinions as follows:

“The four‐step method allows for understanding and technical mastery of skills.”

Quote:

“It is worth using, because it may seemingly take up much time, but in reality it allows you to consolidate your knowledge and skills.”

Students from all the groups said repeatedly: “It's a good method and it should be used more often.”

Another participant of the course expressed his opinion about the new method in the following manner:

“On the other hand, the four‐step method is effective, so there is no question of wasting time—it is an effective use of it.”

#### 3.1.3 Course conditions (atmosphere, room)

“It is great to do the course—no stress at all.”

“The course conditions are good and the instructors are very nice.”

“The course atmosphere is so much low‐key, one can talk—it's so nice.”

#### 3.1.4 Subjective evaluation of the progress of learning

Students commented positively on the method:

“It is great because at the end of the classes I became convinced that I could perform echocardiography on a patient with a severe condition (on my own).”

“The training is effective, because the effects can be seen immediately—you can do the imaging by yourself.”

“You can see the effects, because everyone immediately performs the test on a colleague by themselves.”

“I feel that I am ready to start examining patients.”

#### 3.1.5 Learning in a small group

“It is good that we practice in a small group, because five students per an ultrasound machine are enough.”

“We can say anything in a small group, there is time to ask.”

### 3.2 Room for improvement

The purpose of this study was also to obtain information on the defects of the modified Peyton's four‐step method. The students participating in the elective course decided, however, that “it is difficult to find the negative side of this method.”

#### 3.2.1 The methodological approach

##### Learning through repetition

One of the students thought that “the four‐step method takes time and it may be more difficult for the instructors because they have to perform the same test many times.”

#### 3.2.2 General impressions of the course and the newly introduced four‐step method

One trainee student expressed his doubts as follows: “Why this method anyway?—After all, you can train just as well without it.”

Another participant expressed the following opinion, quote:

“One can always pick on something and find faults in it, but what can be regarded as a disadvantage is the longer time needed to complete all four stages of the method.”

Another student added that the four‐step method “requires constant concentration, which gives headache.”

The trainees said: “I liked it.”

To the question “Is it worthwhile to apply the four‐step method in training imaging, or is it better to selectively use only the second step (which is a slow‐motion demonstration by the instructor, accompanied by the commentary on the activities undertaken) and then to move on smoothly to practical exercises performed by the participants?”, the students responded unanimously that it was worth using.

To the next question: “Why? Is it not a waste of time?”, the participants expressed unambiguously positive opinions about the method used and tried to support it with arguments that multiple repetitions allow for better mastery of the technique and easier memorization.

The students were asked to express their opinion on whether it is worthwhile to continue teaching imaging, not only echocardiographic one, but also other elements of the course with the use of the modified Peyton's four‐step method. According to the participants of the elective class, the method they have learned should be used in teaching practical skills in general. They pointed to the fact that the four‐step method allows you to memorize the correct order in which the test is performed and its technique. During multiple exercises, adequate holding of the head becomes a habit, which is extremely important in proper imaging.

Among the students' opinions, the positive evaluation of Peyton's modified four‐step method was predominant. As it turned out, the time which must be devoted to repeat the examination several times was the only problem. However, according to the students, this was an advantage, because, as the old maxim, well‐known to medical students puts it: “repetitio mater studiorum est”—repetition allows for the consolidation of information and skills.

In the course evaluation questionnaire, the participants wrote: “we need more of such classes” because they are practical and useful for the future career of a doctor. One student stated that it would be best if the participants could start using the skills learned immediately, because otherwise they could forget the technique of performing imaging tests. The participants of the training suggested the course was worth repeating in the future, conducted in a similar way. It would be a good reminder immediately before starting actual professional work, in which the ability to perform imaging studies in emergency cases involving a threat to a patient's life is important.

Everyone answered to the semistructured interview, but only 9 students filled out the questionnaire Table 1.

**Table 1:** Close‐ended questions in questionnaire

Close‐ended questions in questionnaire	Positive answers of the students (≪ yes ≫) (%)
Are you satisfied with the course?	88.9
Do you think the four‐step method is useful in teaching FATE protocol?	77.8
Is the four‐step method difficult?	0

There were collected three negative comments on open‐ended questions:

1. Two students answered: “The method is time‐consuming”
2. One student answered: “Too many steps”

## 4 DISCUSSION

Quantitative analysis of the semistandardized interviews performed with all of the trainees revealed that learning echocardiographic examination using the FATE protocol in the small group by observation and repetition, learning by teaching and active engagement, and the opportunity for independent performance made the modified Peyton's approach a valuable learning and teaching method.

Recent literature in the field of medical education recognizes the four‐step method as a valuable and easy‐to‐use teaching tool. It was also accepted by students.^16^ The modified Peyton's four‐step method is intended for use in small groups. Several studies demonstrated the practical application of the method in teaching difficult medical procedures whose knowledge is essential or obligatory for medical students.^17^, ^18^ Teaching one of the basic medical procedures, which is the insertion of an intravenous catheter by medical students of the first year and second year of study, may be an example here.^15^ The intravenous catheter, commonly referred to as the venous line, should be placed in a patient in the situation necessitating many hours of drugs or fluids administration or repeated intravenous administration of a drug.

In his study, Nikendei^15^ divided a group of nine students to be trained in the intravenous catheter insertion into three smaller groups, assigned to three teachers. The teachers introducing the procedure of intravenous catheter insertion had been instructed how to run the classes so that they complied with the assumptions of the modified Peyton's four‐step method. Then, the teachers and students participating in the study were to share their personal experiences associated with the way the classes were run and assess the practical application of the four‐step method in teaching the insertion of the intravenous catheter.

The trainers and trainees participating in the study were instructed about the stages of Peyton's four‐step method in the following way:

1. The teacher demonstrates the skill at normal pace without comment (the so‐called “Demonstration”)
2. The teacher repeats the procedure, discussing all its phases (called “Deconstruction”)
3. A student explains the individual stages of the procedure, performed again by the teacher (the so‐called Comprehension)
4. A student performs the entire task by him/herself (the so‐called “Performance”)

Subsequently, the participants of the experiment were to perform the procedure first on a dummy and then on a living patient (who received remuneration for the inconvenience and assistance in the realization of the objectives of the study).

Based on the study of Nikendei et al, which was an analysis of partially structured interviews with all the teachers and the students training the insertion of a catheter into a peripheral vein, it has been demonstrated that the modified Peyton's four‐step method is useful and effective. According to the teachers introducing the catheter placement, its advantage lies in the ease of use and the control over the session of intravenous catheter insertion by all students. For students participating in the study, the new method proved to be useful, because according to their statements “they received detailed instructions and were able to repeatedly track the process of the catheter insertion, also in slow motion,” “they weren't annoyed, did not have to ask questions to understand the procedure.”

The study of the implementation of the modified Peyton's four‐step method to teach conducting imaging tests as exemplified by the training in echocardiography in emergency cases according to the FATE protocol confirms the usefulness of the new tool. The teacher running the training echocardiography in emergency cases had direct control over the comprehension and correct implementation of the particular stages of the test by all the trainees.

The Peyton's four‐step method was also used in another study, when teaching a more complex procedure of gastroscopy. Third‐year medical students performed it on a dummy.^19^ Literature describes various modifications of Peyton's method and different ways of instructing the trainees.^20^, ^21^ The usefulness of the method described was demonstrated also in the conditions when tests were conducted on simulated patients.^15^ However, the most widely described Peyton's four‐step method is the one modified with respect to the one teacher per one trainee ratio, where there are several students per one teacher, which could be applied in this study.^16^, ^22^, ^23^ This method has also been used during all ESC (European Society of Cardiology) instructor courses since 2000.^24^, ^25^, ^26^, ^27^

According to the participants of the elective course “Ultrasound imaging in life‐threatening situations,” heart imaging with the use of the FATE protocol is highly difficult. The four‐step method may significantly contribute to the memorization and mastering of a large amount of content in a short time. This is a useful method, which can be successfully used in medical didactics, especially in teaching imaging in emergency cases.

Scientific research indicates that echocardiogram performed using the FATE protocol in patients with a severe condition (referred to in the publication) is a fast and effective method for confirming or excluding cardiac causes for severe, often even critical, condition of the patient.^28^

The students assessed the course and the modified Peyton's method positively.

Based on the partially structured interview with the students, clear conclusions were drawn that the method used during the elective course classes aimed at mastering the skill of echocardiography in emergency cases is recommendable. It is worth using in both the opinions of the participants and the teacher conducting the class, as it organizes presented information and facilitates the acquisition of content which is new for the students. Owing to multiple repetitions, the four‐step method allows for better mastering of the skills introduced during the elective course and the memorization of the test procedure.

The fact that the method used is not wearisome is important. It allows for maintaining vigilance, as each participant is actively involved. The participants of the elective course, who are passive observers at a given moment, are also trying to intently observe the test performed by other participants, as they are aware that they will soon perform it themselves.

The statement “practice makes perfect” is a perfect illustration of the assumptions behind the implementation of the modified four‐step method. After the short course of imaging used in emergency cases, the modified Peyton's four‐step method is proven successful. It makes the course very intense and effective. Students said: “you can't switch off,” “the four‐step method makes you concentrate on the test.”

Due to the small size of the group of students practicing during their elective course in imaging in emergency cases, research should be continued to assess the four‐step method application in teaching echocardiography. The usefulness of the four‐step method in teaching imaging according to other protocols should also be assessed, including standard imaging.

## 5 CONCLUSIONS

Based on the opinions of the participants of the elective course and the teacher conducting the classes, which involved the use of the modified Peyton's four‐step method in teaching echocardiography in emergency cases according to the FATE protocol, it has been determined that the four‐step method is effective in imaging training. It allows for the maximum use of the time assigned to student practice. It consolidates the information and facilitates its memorization through repetition. The authors of the publication decided that it is worthwhile to use the modified four‐step method in teaching imaging, as well as to continue running elective classes in imaging in emergency cases using this method. The study confirms that the modified four‐step method leads to effective learning through observation, the tutor's detailed instruction, several repetitions, and teaching others (Table 2).^15^

**Table 2:** A summary table with a list of positive and negative aspects of the modified Peyton's approach highlighted by the students

Positive aspects of the modified Peyton's approach	Negative aspects of the modified Peyton's approach
“Understandable method”	“The method is time‐consuming”
“Multiple repetitions allow you to master the examination technique”	“Too many steps”
“The four‐step method allows for understanding”	
“The method makes it easier to remember”	
“Concentration on the examination”	
“Teaching others, we learn ourselves, and we remember for longer”	
“It's the effective method”	
“You can see the effects, because everyone immediately performs the examination”	

## CONFLICT OF INTEREST

None declared.

## AUTHOR CONTRIBUTIONS

AS contributed to study conception and design. MN supervised the study. AS and TG contributed to data entry. PK, MP, KJ, MSz, and GC helped data analysis and interpretation. AS drafted the manuscript. AS contributed to discussion and manuscript revision. All the coauthors approved the final version of the manuscript.

## ETHICS APPROVAL

The Ethics Committee of the Jagiellonian University accepted the study protocol (No 1072.6120.164.2017).

## PROVENANCE AND PEER REVIEW

Not commissioned; externally peer reviewed.

## DATA SHARING STATEMENT

A fully anonymous data set is available from the authors (agnieszka.skrzypek@gmail.com) on request.
